# Transarterial Chemoembolization in Treatment-Naïve and Recurrent Hepatocellular Carcinoma: A Propensity-Matched Outcome and Risk Signature Analysis

**DOI:** 10.3389/fonc.2021.662408

**Published:** 2021-06-04

**Authors:** Yiming Liu, Yanqiao Ren, Sangluobu Ge, Bin Xiong, Guofeng Zhou, Gansheng Feng, Songlin Song, Chuansheng Zheng

**Affiliations:** ^1^ Department of Radiology, Union Hospital, Tongji Medical College, Huazhong University of Science and Technology, Wuhan, China; ^2^ Hubei Provinve Key Laboratory of Molecular Imaging, Wuhan, China

**Keywords:** hepatocellular carcinoma, transarterial chemoembolization, liver resection, recurrence, propensity score matching

## Abstract

**Objectives:**

The purpose of this study was to evaluate the efficacy and safety of transarterial chemoembolization (TACE) in the treatment of patients with treatment-naïve hepatocellular carcinoma (TN-HCC) and recurrent HCC (R-HCC). In addition, risk signature analysis was performed to accurately assess patients’ recurrence and survival.

**Methods:**

This retrospective study assessed the consecutive medical records of TN-HCC and R-HCC patients from January 2014 to December 2018. In order to reduce the patient selection bias, propensity score matching (PSM) analysis was applied. Conditional inference tree was used to establish a risk signature.

**Results:**

A total of 401 eligible patients were included in our study, including 346 patients in the TN-HCC group and 55 patients in the R-HCC group. Forty-seven pairs of patients were chosen after the PSM analysis. Before the PSM analysis, the objective tumor regression (ORR) and disease control rate (DCR) of R-HCC patients were better than that of TN-HCC patients; however, after the PSM analysis, there was no significant difference in the ORR and DCR between the two groups (P>0.05). Before the PSM analysis, the median overall survival (OS) and progression-free survival (PFS) in the R-HCC group were significantly greater than those of the TN-HCC group (OS: 24 months *vs.* 18 months, *P* =0.004; PFS: 9 months *vs.* 6 months, *P* =0.012). However, after the PSM analysis, the median OS and PFS in the R-HCC group were inferior to those in the TN-HCC group (OS: 24 months *vs.* 33 months, *P*= 0.0035; PFS: 10 months vs. 12 months, *P* = 0.01). The conditional inference tree divided patients into different subgroups according to tumor size, BCLC stage, and TACE sessions and shared different hazards ratio to recurrence or survival.

**Conclusion:**

Patients with R-HCC treated with TACE achieved satisfactory results, although survival after the PSM analysis was not as good as in the TN-HCC group. In addition, risk signature based on conditional inference tree analysis can more accurately predict the recurrence and survival in both groups of patients.

## Introduction

Hepatocellular carcinoma (HCC) is the fifth most common cancer and one of the most frequent causes of cancer-related death (1). Globally, and especially in China, the prognosis of HCC patients remains a depressing issue. Currently, therapies such as liver resection (LR) and liver transplantation have the potential to cure patients with preserved liver function, but these curative therapies only benefit a quarter of HCC patients (2, 3). In addition, intrahepatic recurrence and *de novo* tumor emergence in the liver remnant after LR are common, with a 5-year recurrence rate of up to 70%-80% (4). Although this is a common clinical manifestation, there is still no consensus on the treatment of recurrent HCC (R-HCC) after LR, which remains a thorny issue that currently confounds clinicians and patients.

When intrahepatic tumors recur, re-resection or salvage liver transplantation remains the best way to cure the patient. However, not all recurrent patients are eligible for surgical treatment due to the limited reserve of liver function in the residual liver, postoperative adhesion, or lack of a liver donor (5, 6). As a result, only a small number of patients benefit from curative treatments, which may create an incentive to try other therapies and approaches.

Transarterial chemoembolization (TACE) combines targeted chemotherapy with arterial embolization, which is the main palliative method for the treatment of HCC (7). Two randomized controlled trials (2, 8) established the status of TACE in BCLC stage B HCC patients, for whom TACE is recommended as the standard of care. Meanwhile, TACE has also been reported in patients with BCLC stage C HCC, and the results indicated that TACE can benefit these patients (9, 10). Currently, most studies have assessed the efficacy of TACE in patients with treatment-naïve HCC (TN-HCC), but it is also worth exploring whether TACE can benefit patients with R-HCC after LR compared with patients with TN-HCC.

Since TACE is not limited by tumor size, location and number of lesions, it is suitable for most types of HCC and is widely used in patients with R-HCC after LR (11). Therefore, the purpose of this study was to evaluate the efficacy and safety of TACE in patients with TN-HCC and R-HCC after LR by propensity score matching (PSM) analysis. Furthermore, prognostic factors influencing the efficacy of TACE in both groups were also analyzed. Meanwhile, the conditional inference tree analysis was constructed to assess recurrence and survival in both groups after TACE.

## Methods

### Study Design and Patient Selection

We reviewed the electronic medical records of 2158 consecutive patients who received TACE in our medical center from January 2014 to December 2018 for HCC, including patients with TN-HCC and with R-HCC after LR. Prior to these patients received initial TACE, the treatment plan was nominated by the multidisciplinary tumor board. This retrospective study was approved by the institutional review board of the Union Hospital, Tongji Medical College, Huazhong University of Science and Technology. Written informed consent for the patients’ data to be used for research purposes was obtained from all patients prior to treatment.

**Figure 1:**
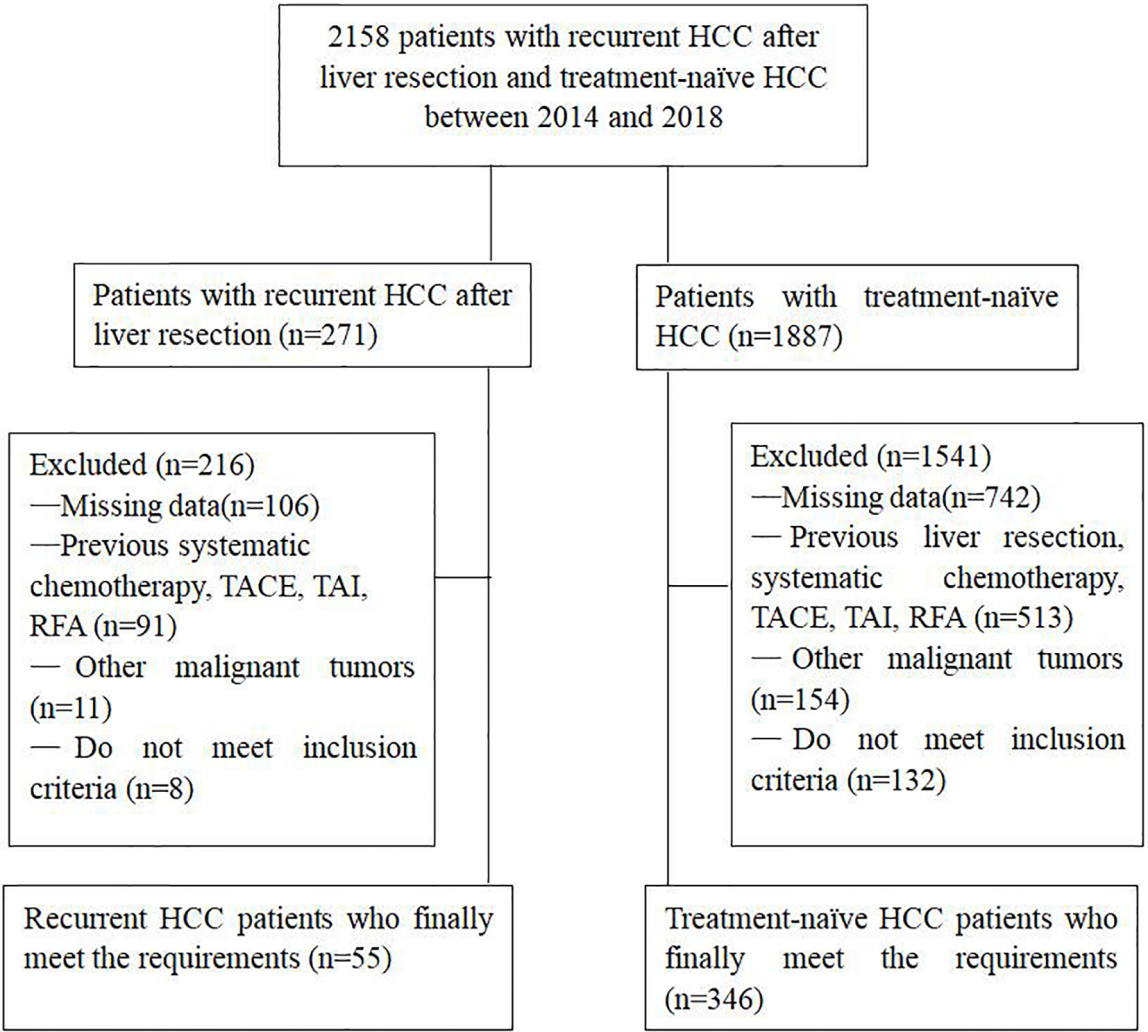
Flow chart shows the screening procedure for patients with recurrent HCC after liver resection and treatment-naïve HCC.

The diagnosis of HCC depended on the guidelines of the European Association for the Study of Liver and the American Association for the Study of Liver Disease (12). A total of 401 patients in this study met the inclusion criteria: (1) age > 18 years; (2) Child-Pugh class A or B; (3) Eastern Cooperative Oncology Group (ECOG) performance status of 0–1. The exclusion criteria were: (1) Incomplete clinical information; (2) main portal vein obstruction; (3) BCLC stage D; (4) ECOG>1; (5) Severe medical comorbidities, including hepatic dysfunction (total bilirubin serum levels > 3 mg/dL, serum albumin level < 2.0 mg/dL, INR > 1.5), renal impairment (serum creatinine level >2 mg/dL) and severe coagulation disorders (prothrombin activity<40% or platelet count<30X10^9^/L); (6) Uncontrolled infection (**Figure 1**).

### TACE Procedure

TACE was performed based on our institutional standard protocol and has been described previously (13, 14). Briefly, angiography was performed to determine tumor staining and tumor-supplying vessels, and a 5-F catheter (Cook, Bloomington, Indiana, USA) or 3-F microcatheter (Progreat, Terumo, Tokyo, Japan) was inserted as far as possible into the tumor supplying vessels. Then, an emulsion of 2–20 mL iodized oil (Lipiodol Ultra-Fluid; Laboratoire Andre Guerbet, Aulnay-sous-Bois, France) and 20–60 mg doxorubicin hydrochloride was injected into the target vessels. Finally, gelatin sponge particles (300–700μm, Alicon, Hangzhou, China) were injected for additional embolization until the stasis of arteries flow was achieved. After embolization, reexamination angiography of the feeding artery was performed to confirm the devascularization.

### Definition and Evaluation of Data

Overall survival (OS) and progression-free survival (PFS) were compared between TN-HCC and R-HCC groups. OS referred to the time from the initial TACE procedure to death or last follow-up. PFS was defined as the interval between the date of the first TACE procedure and the date of progression for patients who displayed radiologic evidence of disease progression or the date of death or last follow-up. Modified Response Evaluation Criteria in Solid Tumors was used to assess tumor response 1 month after initial TACE. Objective tumor regression (ORR) referred to complete response (CR) or partial response (PR). Disease control rate (DCR) represented CR, PR or stable disease (SD). The safety of TACE was evaluated by the Society of Interventional Radiology classification system (15). Those complications that lead to death and disability were defined as major complications that significantly increase the level of care or extend the length of hospital stay. Also, complications such as fever, vomiting and so on were considered minor.

Early recurrence was defined as a time interval of less than 2 years from curative LR to tumor recurrence, and a time interval of more than 2 years was considered as late recurrence. Curative LR meant that all tumor nodules were completely removed, the resection margin was clean, histological examination showed that there was no tumor on the cut surface, and no residual cancer in liver remnants was examined by abdominal contrast-enhanced computed tomography (CT) or magnetic resonance (MR) 1 month after the surgery (16).

### Follow-Up and Repeated TACE

All patients were followed up 6-8 weeks after initial TACE. Follow-up evaluations included laboratory tests (including hematology and biochemical analyses) and abdominal contrast-enhanced CT or MR. Repeated TACE was performed in patients with residual viable or recurrent tumor in the liver on contrast-enhanced CT or MR imaging and with preserved liver function. If tumors were completely necrotic, abdominal contrast-enhanced CT or MR imaging and laboratory examination were performed every 2-3 months. Patients were followed until death or the end point of the study (December 31, 2020).

### PSM Analysis

To reduce the patient selection bias and balance the variables between TN-HCC and R-HCC patients, a balanced cohort was assembled using a PSM analysis with a 1:1 ratio, and the value of the caliper was 0.05. The baseline variables including age, gender, Child–Pugh class, BCLC stage, tumor size, tumor number, TACE sessions, HBV infection, platelet, alanine aminotransferase, aspartate aminotransferase, prothrombin activity, total bilirubin, alpha-fetoprotein (AFP) level, and albumin were matched in our model.

### Statistical Analysis

Discrete variables were represented by numbers with percentages and were calculated by Chi-square test, and continuous variables were presented as mean ± standard deviation and were calculated by Student’s t-test. Kaplan-Meier method was used to evaluate the differences of PFS and OS between the two groups. The 95% confidence interval (CI) was calculated for median OS, median PFS, and hazard ratio (HR). A Cox proportional hazard regression model was used to analyze the potential prognostic factors affecting OS and PFS. Potential risk factors identified in univariate Cox model (*P*<0.1) were then entered into the multivariate Cox model. Conditional inference trees were constructed to further evaluate the association between RFS/OS and the associated risk factors. All analyses were performed using R (Version 3.6.1; R Foundation for Statistical Computing, Vienna, Austria; https://www.r-project.org/) and RStudio (Version 1.2.1335; RStudio, Inc., Boston, MA; https://www.rstudio.com/). All statistical tests were two-tailed, with *P* < 0.05 indicating a significant difference.

## Results

### Study Population and Patient Characteristics

From January 2014 to December 2018, a total of 401 patients were included in our study, including 346 TN-HCC patients and 55 patients with R-HCC. Before the initial TACE, the mean tumor size of R-HCC patients was significantly smaller than that of the TN-HCC patients (*P*<0.001), and there were significant differences in BCLC stage, Child-Pugh class, alanine transaminase, and aspartate aminotransferase between the two groups (*P*<0.05). In addition, there was no significant difference in the other baseline characteristics between the two groups. Baseline demographics and characteristics of the 401 patients are shown in **Table 1**. The median follow-up duration was 18.0 months (range, 2–69 months) in the TN-HCC group and 22.0 months (range, 4–71 months) in the R-HCC group. At the end of follow-up, 226 (65.3%) patients in the TN-HCC group and 31 (56.4%) patients in the R-HCC group died.

**Table 1:** Baseline characteristics of patients between the two groups before and after PSM analysis.

Characteristics	Before PSM (No, %; Mean±SD)		*P* value	After PSM (No, %; Mean±SD)		*P* value
	TACE for R-HCC (n=55)	TACE for TN-HCC (n=346)		TACE for R-HCC (n =47)	TACE for TN-HCC (n =47)	
Age (years)	52.7±10.48	55.14±11. 63	0. 136	54.43±12.21	54. 64±9. 09	
Gender			0. 808			0. 924
Male	46 (83 6%)	281 (81.2%)		40 (81.6%)	40 (81.6%)	
Female	9 (16.4%)	65 (18.8%)		7 (14.9%)	7 (14.9%)	
ECOG performance	** **	** **	0.778			1
0	44 (80%)	271 (78.3%)		40 (81.6%)	40 (81.6%)	
1	11 (20%)	75 (21.7%)		7 (14.9%)	7 (14.9%)	
Child-Pugh class	** **		0. 016			1
A	53 (96.4%)	291 (84.1%)		45 (95.7%)	45 (95.7%)	
B	2(3.6%)	55 (15.9%)		2 (4.3%)	2 (4.3%)	
BCLC stage			<0. 001			0. 869
A	4 (7.3%)	48 (13.9)		4 (8.5%)	4 (8.5%)	
B	43 (78.2%)	168 (48.6)		33 (70.2%)	35 (74.5%)	
C	8 (14.5%)	130 (37.6)		10 (21.3%)	8 (17.0%)	
HBV infection	** **		0.448			0.55
Yes	47 (85.5%)	277 (80.1%)		42 (89.4%)	39 (83.0%)	
No	8 (14.5%)	69 (19.9%)		5 (10.6%)	8 (17%)	
AFP (ng/m,)			0. 645			0. 822
>400	18 (32.7%)	128 (37%)		15 (31.9%)	13 (27.7%)	
≤400	37 (67.3%)	218 (63%)		32 (68.1%)	34 (72.3%)	
ALT( IU/L)	33.20±20.69	60.40±86.13	0. 02	35.47±19.44	33.21±21.83	0.598
AST ( IU /L)	3816±42.60	69.21±80.05	0. 005	49.00±45.58	39.57±45. 93	0. 321
Total bilirubin (μmol/L)	96.11±76.81	99.87±63.45	0.692	93.23±61.72	93.89±77.84	0.964
Platelet count(109/L)	139.85 (70.40)	152.64 (88.87)	0.31	131.83±69.38	141.85±74.38	0.501
Albumin (g/dL)	38.88± 3.89	36.39±5.64	0.515	39.31±5.11	38.96± 4.01	0. 712
Prothrombin time, INR	14.05±1.39	14.24±1.49	0. 354	14.11±0.92	14. 06±1.49	0. 848
Number of tumors	** **		0. 144			>0. 999
1	13 (23.6%)	120 (34.7%)		13 (27.7%)	12 (25.5%)	
>1	42 (76.4%)	226 (65.3%)		34 (72.3)	35(74.5)	
Maximal tumor diameter (cm)	2. 96±1.71	7.42±4. 64	<0. 001	3.16±2. 17	3.09±1.73	0. 879
TACE sessions	9. 13+3.07	8.50+3.56	0.22	8.89±3.57	9.17+3.30	0. 697

PSM, propensity score matching; SD, standard deviation; R-HCC, recurrent hepatocellular carcinoma; TN-HCC, treatment-naïve hepatocellular carcinoma; ECOG, Eastern Cooperative Oncology Grou, BCLC, Barcelona Clinic Liver Cancer; HBV, hepatitis B; AFP, alpha-fetoprotein; ALT, alanine transaminase; AST, aspartate aminotransferase; TACE, transarterial chemoembolization.

### Complications or Adverse Events

In TN-HCC group, 7 patients (2%) had serious complications. Three patients presented with biloma and four with liver abscess, and their symptoms improved gradually through percutaneous bile duct or abscess drainage. In R-HCC group, 1 patient (1.8%) developed biloma, and the symptom was improved by percutaneous bile duct drainage. There was no significant difference in the incidence of major complications between the two groups. Common minor complications such as fever, nausea and vomiting, abdominal pain, abnormal liver function, and scanty ascites occurred in 96 patients (27.7%) in TN-HCC group and 12 patients (21.8%) in R-HCC group.

### Efficacy Comparison Between the Patients of TN-HCC and R-HCC

The morphological response of the target lesion was verified by abdominal contrast-enhanced CT or MR imaging. The ORR of TN-HCC patients was 61.6%, and that of R-HCC patients was 76.4% (*P*=0.034). In addition, the DCR of TN-HCC patients was 81.5%, and that of R-HCC patients was 90.9% (*P*=0.086). Hence, compared with TN-HCC patients, R-HCC patients had better ORR.

**Figure 2:**
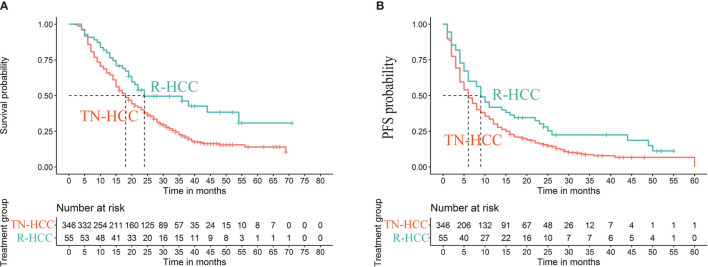
Kaplan-Meier curves of cumulative survival **(A)** and progression-free survival (PFS) **(B)** in patients with recurrent HCC after liver resection and treatment-naïve HCC before propensity score matching.

Median OS was 18 months (95% CI 16 months, 20 months) in the TN-HCC group and 24 months (95% CI 19 months, 54 months) in the R-HCC group (*P*=0.004) (**Figure 2A**). Median PFS was 6 months (95% CI 5 months, 7 months) in the TN-HCC group and 9 months (95% CI 6 months, 16 months) in the R-HCC group (P =0.012) (**Figure 2B**).

### PSM Analysis

As baseline characteristics of TN-HCC patients were different from those of R-HCC patients, a PSM analysis was performed. After the PSM analysis, 47 pairs were selected (**Table 1**). The ORR of TN-HCC patients was 78.7%, and that of R-HCC patients was 72.3%, with no statistical difference between the two groups (*P*=0.472). Similarly, there was no statistically significant difference in DCR between the two groups (91.5% *vs* 87.2%, *P*=0.503).

**Figure 3:**
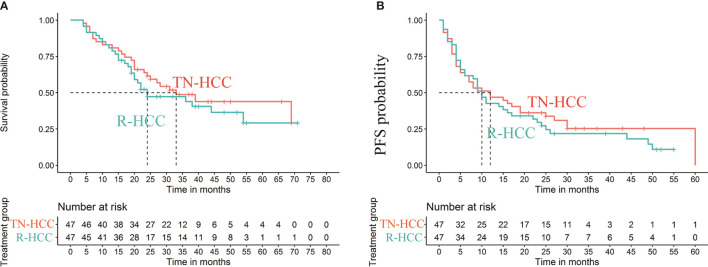
Kaplan-Meier curves of cumulative survival **(A)** and progression-free survival (PFS) **(B)** in patients with recurrent HCC after liver resection and treatment-naïve HCC after propensity score matching.

The median OS in the TN-HCC group and the R-HCC group were 33 months (95% CI, 23-) and 24 months (95% CI, 19–54), respectively, and the difference between the two groups was significantly different (P= 0.0035) (**Figure 3A**). Multivariable analysis indicated that BCLC C and hepatitis B were independent risk factors for OS, while TACE sessions were associated with better OS (**Table 2**).

**Table 2:** Univariate and multivariate analysis of prognostic factors for overall survival (OS) after PSM analysis.

Variable	Univariate analysis		Multivariate analysis	
	HR(95%CI)	*P* value	HR(95%CI)	*P* value
Age	0.996 (0.972~1.020)	0.7208		
Sex		0.6777		
Male	Reference			
Female	1.165 (0.567~2.393)			
Number of tumors		0.0254		0.16
1	Reference		Reference	
>1	0.525 (0.299~0.924)		0.631 (0.332~1.199)	
HBV infection		0.3032		
No	Reference			
Yes	1.570 (0.665~3.705)			
Child-Pugh class		0.5148		
A	Reference			
B	1.603 (0.387~6.637)			
BCLC stage				
A	Reference		Reference	
B	0.438 (0.191~1.005)	0.0514	0.948 (0.347~2.593)	0.9178
C	1.711 (0.686~4.267)	0.2496	2.814 (1.051~7.535)	0.0396
AFP (ng/ml)				
≤400	Reference		Reference	
>400	1.693 (0.962~2.980)	0.0678	1.953 (1.092~3.493)	0.024
TACE sessions	0.887 (0.824~0.955)	0.0015	0.890 (0.818~0.968)	0.0068
Maximal tumor diameter (cm)	1.056 (0.917~1.215)	0.449		
Platelet count (10^9^/L)	1.003 (0.999~1.007)	0.1947		
ALT(IU/L)	1.003 (0.990~1.017)	0.6313		
AST (IU/L)	1.000 (0.994~1.006)	0.9553		
Albumin (g/dL)	0.960 (0.904~1.021)	0.1919		
Total bilirubin (µmol/L)	1.002 (0.998~1.005)	0.415		
Prothrombin time, INR	0.961 (0.744~1.242)	0.7616		
Group				
TN-HCC	Reference			
R-HCC	1.243 (0.723~2.138)	0.4311		

PSM, propensity score matching; HR, hazard ratio; CI, confidence interval; BCLC, Barcelona Clinic Liver Cancer; HBV, hepatitis B; AFP, alpha-fetoprotein; ALT, alanine transaminase; AST, aspartate aminotransferase; TACE, transarterial chemoembolization; TN-HCC, treatment-naïve hepatocellular carcinoma; R-HCC, recurrent hepatocellular carcinoma.

Median PFS was 12.0 months (95% CI: 6 months, 60 months) in the TN-HCC group and 10.0 months (95% CI: 4 months, 25 months) in the R-HCC group (*P* = 0.01) (**Figure 3B**). Univariate analyses showed that AFP level and platelet were significantly associated with PFS (**Table 3**), but there was no independent risk factor in multivariate analyses for PFS.

**Table 3:** Univariate and multivariate analysis of prognostic factors for progression-free survival (PFS) after PSM analysis.

Variable	Univariate analysis		Multivariate analysis	
	HR (95%CI)	*P* value	HR (95%CI)	*P* value
Age	1.000 (0.979~1.022)	0.9847		
Sex		0.5752		
Male	Reference			
Female	1.187 (0.651~2.165)			
Number of tumors		0.3973		
1	Reference			
>1	0.803 (0.483~1.334)			
HBV infection		0.4748		
No	Reference			
Yes	1.276 (0.654~2.489)			
Child-Pugh class		0.7022		
A	Reference			
B	1.254 (0.393~4.002)			
C				
BCLC stage				
A	Reference			
B	0.704 (0.318~1.559)	0.387		
C	1.290 (0.521~3.190)	0.5818		
AFP (ng/ml)		0.0313	1.591 (0.955~2.651)	0.0744
≤400	Reference			
>400	1.735 (1.051~2.866)			
TACE sessions	0.975 (0.909~1.046)	0.4842		
Maximal tumor diameter (cm)	1.027 (0.907~1.164)	0.6711		
Platelet count (10^9^/L)	1.003 (1.000~1.007)	0.0488	1.003(0.999~1.006)	0.1187
ALT(IU/L)	0.999 (0.987~1.011)	0.88		
AST (IU/L)	1.000 (0.995~1.005)	0.9608		
Albumin (g/dL)	0.993 (0.944~1.044)	0.7742		
Total bilirubin (µmol/L)	0.999 (0.996~1.002)	0.5925		
Prothrombin time, INR	0.951 (0.770~1.175)	0.6426		
Group				
TN-HCC	1.142 (0.718~1.816)	0.5756		
R-HCC				

PSM, propensity score matching; HR, hazard ratio; CI, confidence interval; BCLC, Barcelona Clinic Liver Cancer; HBV, hepatitis B; AFP alpha-fetoprotein; ALT, alanine transaminase; AST, aspartate aminotransferase; TACE, transarterial chemoembolization; TN-HCC, treatment-naïve hepatocellular carcinoma; R-HCC, recurrent hepatocellular carcinoma.

### Decision Tree Model and Subgroup Analysis

**Figure 4:**
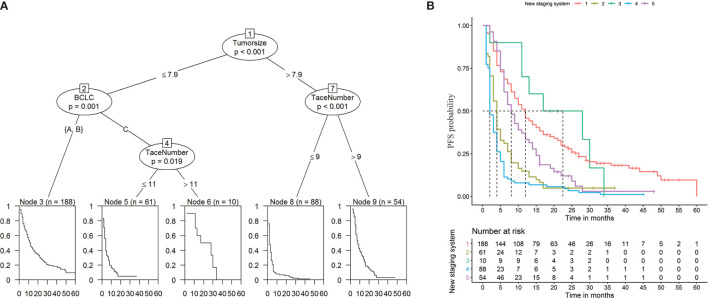
Prediction of progression-free survival (PFS) **(A)** and Kaplan-Meier curves of PFS **(B)** based on decision tree results.

**Figure 5:**
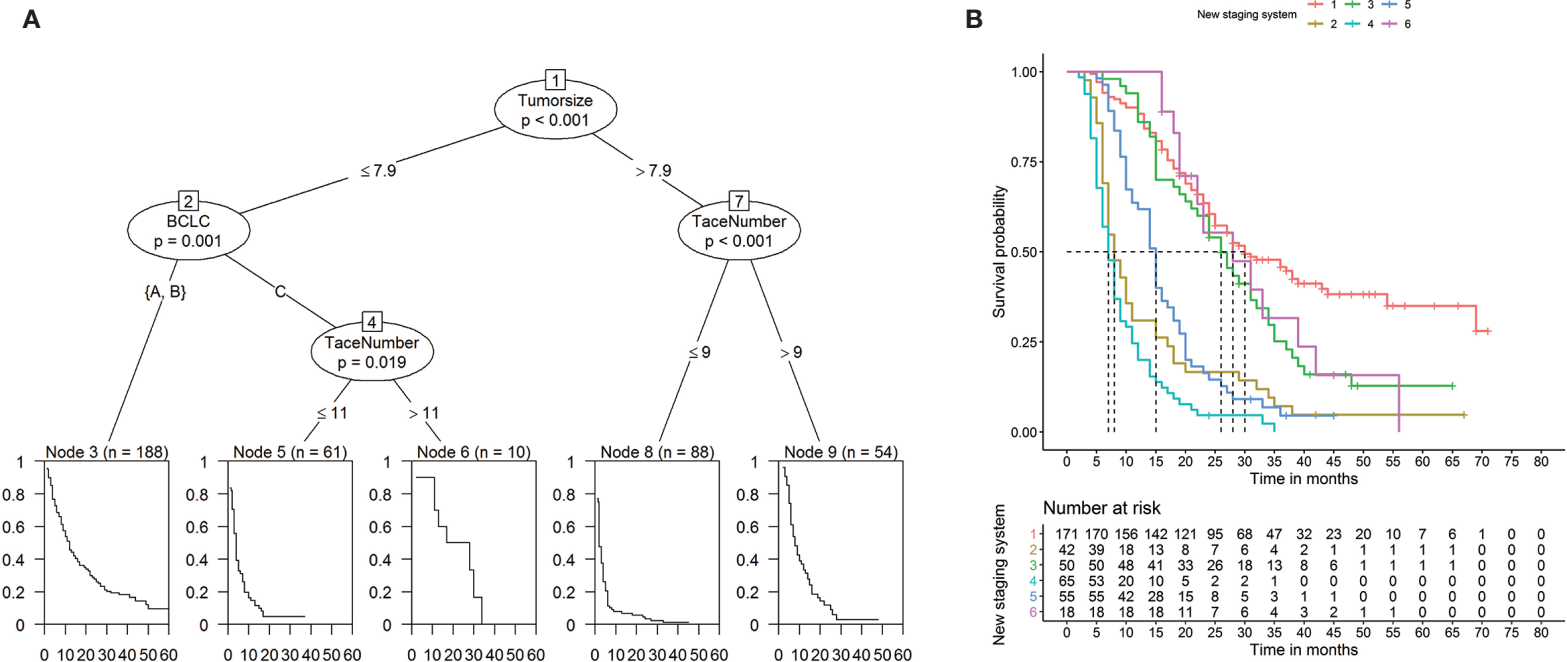
Prediction of overall survival (OS) **(A)** and Kaplan-Meier curves of OS **(B)** based on decision tree results.

To establish a risk signature that can classify patients into homogeneous subpopulations according to PFS and OS, we further constructed the conditional inference tree analysis using PFS and OS as predictive endpoints, respectively. After pruning the decision trees using the postpruning method, 5 terminal nodes (subpopulations) representing a recurrence signature were identified (**Figures 4A, B**). Furthermore, 6 subgroups representing a survival signature were identified (**Figure 5A, B**). Patients entered into different subgroups according to tumor size, BCLC stage, and TACE sessions and shared different hazards ratio to recurrence or survival (**Table 4**).

**Table 4:** The Cox regression analysis of progression-free survival (PFS) or overall survival (OS) according to new stage.

Categorical variable	HR (95%CI)	*P* value
PFS		
1 Tumor size <=7.9 & BCLC =“A/B”	Reference	
2 Tumor size <=7.9 & BCLC=“C”& Number of TACE <=11	2.68 (1.97, 3.65)	<0.001
3 Tumor size<=7.9 & BCLC=“C”)& Number of TACE >11	0.80 (0.39, 1.64)	0.546
4 Tumor size >7.9 & Number of TACE <=9	3.96 (3.02, 5.21)	<0.001
5 Tumor size >7.9 & Number of TACE >9	1.48 (1.07, 2.04)	0.018
OS		
1 BCLC =“A/B”& Tumor size <=6.9	Reference	
2 BCLC =“A/B”& Tumor size > 6.9 & Number of TACE <=8	4.35 (2.99, 6.31)	<0.001
3 BCLC =“A/B”& Tumor size > 6.9 & Number of TACE >8	1.57 (1.09, 2.27)	0.016
4 BCLC =“C”& Number of TACE <= 8	8.37 (5.98, 11.72)	<0.001
5 BCLC =“C”& 11>=Number of TACE > 8	3.50 (2.48, 4.95)	<0.001
6 BCLC =“C”& Number of TACE >11	1.36 (0.76, 2.42)	0.305

HR, hazard ratio; CI, confidence interval; BCLC, Barcelona Clinic Liver Cancer; TACE, transarterial chemoembolization.

## Discussion

In this study, compared with R-HCC patients, TN-HCC patients showed poor baseline characteristics at the time of the first TACE. Accordingly, the results of our study indicated that patients in the R-HCC group had better tumor response, OS and PFS than patients in the TN-HCC group before the PSM analysis. However, after PSM, patients in the TN-HCC group had better OS and PFS than patients in the R-HCC group, which further indicates that the recurrence of tumor after LR leads to unsatisfactory long-term survival and thus death of HCC patients (16, 17).

So far, intrahepatic recurrence remains a thorny problem, and the choice of treatment after recurrence is extremely important. For patients with recurrence, resection or ablation is the optimal therapeutic option, provided that the liver function of these patients is Child-Pugh class A or B, adequate liver reserve, and appropriate tumor location, etc. (18). If these conditions are not met, TACE may be the treatment of choice. In our study, patients were eligible to receive TACE because most had multiple recurrent tumors or inadequate liver reserve or the tumor location was unsuitable for ablation. Nevertheless, the 1, 3-year OS rates in recurrent patients treated with TACE in this study were not inferior to the 1, 3-year OS rates reported in patients undergoing repeat resection or ablation. It has been reported that the 1- and 3-year OS rates of patients with recurrent HCC after LR were 71–94% and 41–75% (19–21), respectively, while the 1- and 3-year OS rates of patients undergoing radiofrequency ablation were 82% and 47-54% (22, 23), respectively. Similar to these reports, the 1 - and 3-year OS rates in our study after PSM were 80.9% and 43.9%, respectively.

At the same time, this study compared the efficacy between the two groups of TN-HCC patients treated with TACE and those patients with R-HCC. Currently, TACE has been recognized as the standard method for unresectable HCC patients and a significant number of studies have confirmed the therapeutic effect of TACE on TN-HCC patients (3, 24). However, to date, few studies (25) have reported the outcomes of TACE for R-HCC patients. Therefore, this study compared the therapeutic effects of TACE on the two groups of patients, and the results demonstrated that OS and PFS of R-HCC patients were slightly inferior to TN-HCC patients after PSM analysis. Hence, based on the results of PSM analysis, we believe that early dynamic detection of R-HCC can significantly improve the prognosis of patients.

Recurrence and survival after TACE in both groups are critical to the prognosis of patients. Zhuang et al. (26) incorporated seven prognostic factors to construct a prognostic nomogram, and concluded that TACE combining with RFA was beneficial in patients with recurrent HCC in the low-risk group after LR, while TACE alone was sufficient for patients in the medium/high-risk group. Meanwhile, Lu et al. (27) retrospectively analyzed clinical data from 597 HCC patients treated with TACE, suggesting that elevated platelet was associated with poor survival in HCC patients. In our study, in order to establish a risk signature that divides patients into homogeneous subgroups according to PFS and OS, the conditional inference tree analysis were constructed. Then, the prognosis of the two groups of patients was accurately determined according to the tumor diameter, BCLC stage and TACE sessions of patients.

Our study indicated that TACE procedure was well tolerated in patients with TN-HCC or R-HCC, and the 2% serious complication rate increases the number of literatures (28, 29) supporting chemoembolization as a safe method. In this study, the symptoms of patients with biloma and liver abscess were gradually improved after percutaneous drainage. Similar to other studies (30–33), postembolism syndrome such as fever, vomiting, and abdominal pain were the most common complications in the current study, and most of them are self-limiting.

This study had certain limitations. Retrospective and non-randomized design is one of the limitations. Although the PSM analysis was applied, there is still the risk of selection bias. In addition, the data in this study came from a single-center with a small sample size. Therefore, an adequately powered multi-center prospective randomized controlled trial is necessary to verify our results.

In conclusion, patients with R-HCC treated with TACE achieved satisfactory results, although survival after PSM was not as good as in the TN-HCC group. In addition, the conditional inference tree was used to construct a risk signature that divides patients into homogeneous subgroups according to PFS and OS, which can more accurately predict the prognosis of patients in the two groups.

## Data Availability Statement

The raw data supporting the conclusions of this article will be made available by the authors, without undue reservation.

## Ethics Statement

The studies involving human participants were reviewed and approved by Union Hospital, Tongji Medical college, Huazhong University of Science and Technology. The patients/participants provided their written informed consent to participate in this study.

## Author Contributions

YL, YR, and LB collected the patients’ data. YL and YR drafted the manuscript. BX, GZ, GF, SS, and CZ revised the manuscript. YL, YR, LB, and SS analyzed and interpreted the data. BX and GZ made substantial contributions to the conception of the work. SS and CZ made substantial contributions to the design of the work and have revised the manuscript substantively. All authors contributed to the article and approved the submitted version.

## Funding

This work was supported by grant from National Nature Science Foundation of China (81873919 and 81801810), Free Innovation Pre-research Fund of Union Hospital, Tongji Medical College, Huazhong University of Science and Technology (02.03.2019-157).

## Conflict of Interest

The authors declare that the research was conducted in the absence of any commercial or financial relationships that could be construed as a potential conflict of interest.
